# Current status of contemporary diagnostic radiotracers in the management of breast cancer: first steps toward theranostic applications

**DOI:** 10.1186/s13550-023-00995-2

**Published:** 2023-05-17

**Authors:** Renske Altena, Antonios Tzortzakakis, Siri Af Burén, Thuy A. Tran, Fredrik Y. Frejd, Jonas Bergh, Rimma Axelsson

**Affiliations:** 1 Institutionen Oncology-Pathology, Karolinska Institutet, Stockholm, Sweden; 2 Medical Unit Breast, Endocrine Tumors and Sarcoma, Theme Cancer, Karolinska Comprehensive Cancer Center, Karolinska University Hospital, Solna, Sweden; 3 Division of Radiology, Department for Clinical Science, Intervention and Technology (CLINTEC), Karolinska Institutet, Stockholm, Sweden; 4 Medical Radiation Physics and Nuclear Medicine, Functional Unit of Nuclear Medicine, Karolinska University Hospital, Huddinge, Sweden; 5 Department of Radiopharmacy, Karolinska University Hospital, Solna, Sweden; 6 Department of Immunology, Genetics and Pathology, Uppsala University, Uppsala, Sweden; 7 Affibody AB, Solna, Sweden; 8 Department of Molecular Medicine and Surgery, Karolinska Institutet, Stockholm, Sweden

**Keywords:** Molecular imaging, Predictive biomarker, Breast cancer

## Abstract

**Background:**

Expanding therapeutic possibilities have improved disease-related prospects for breast cancer patients. Pathological analysis on a tumor biopsy is the current reference standard biomarker used to select for treatment with targeted anticancer drugs. This method has, however, several limitations, related to intra- and intertumoral as well as spatial heterogeneity in receptor expression as well as the need to perform invasive procedures that are not always technically feasible.

**Main body:**

In this narrative review, we focus on the current role of molecular imaging with contemporary radiotracers for positron emission tomography (PET) in breast cancer. We provide an overview of diagnostic radiotracers that represent treatment targets, such as programmed death ligand 1, human epidermal growth factor receptor 2, polyadenosine diphosphate-ribose polymerase and estrogen receptor, and discuss developments in therapeutic radionuclides for breast cancer management.

**Conclusion:**

Imaging of treatment targets with PET tracers may provide a more reliable precision medicine tool to find the right treatment for the right patient at the right time. In addition to visualization of the target of treatment, theranostic trials with alpha- or beta-emitting isotopes provide a future treatment option for patients with metastatic breast cancer.

## Background

Breast cancer is the most frequently diagnosed cancer in women worldwide [1]. Rapidly expanding therapeutic possibilities have increased early and metastatic breast cancer management opportunities.

Drugs targeting programmed death ligand 1 (PD-L1, e.g., atezolizumab and pembrolizumab), the human epidermal growth factor receptor 2 (HER2, e.g., trastuzumab deruxtecan and tucatinib) and polyadenosine diphosphate-ribose polymerase (PARP, olaparib and talazoparib) have within the past few years gained new indications for specific subgroups of patients with breast cancer [2–7]. Moreover, already existing indications for drugs targeting estrogen receptors (ER) and HER2 have been consolidated.

Patient selection for the treatment with these drugs is currently made by molecular analyses, using immunohistochemistry (IHC) and/or in situ hybridization (ISH), on a tumor biopsy, from either the (archived) primary tumor or a metastatic lesion. However, up- and downregulation has been described upon progression from primary to metastatic tumors and during clonal progression in metastatic disease. For example, PD-L1 expression is lower in metastatic versus primary disease [8], and ER and HER2 expression can change during tumor progression [9]. For this reason, international guidelines recommend biopsy-based strategies during breast cancer disease progression [10].

**Fig. 1:**
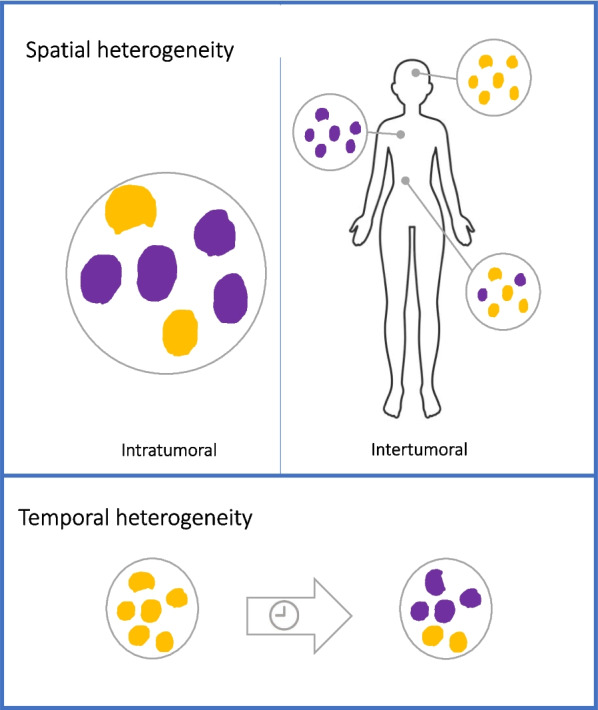
Different causes of heterogeneity in molecular features of cancers, both in relation to differences in expression within and between disease localizations, and changes in expression over time

Assessment of predictive biomarkers on a tumor biopsy has its limitations—invasiveness with needs for tissue samples which is not always practically feasible; non-representativeness of a solitary biopsy for the whole body because of intra- and intertumoral as well as spatial heterogeneity in receptor expression; and limited efficacy in predicting whether the administered drugs will reach their target and thereby can execute anticancer activity (Fig. 1). Therefore, access to more refined diagnostic tools that could provide information about the presence of the target for such treatments with high reliability is critical.

Positron emission tomography (PET) imaging with receptor-specific tracers can provide, in a non-invasive manner, information on whether the target for treatment exists. At the same time, PET enables quantification of the target in the whole body in real time and with high accuracy. In addition to diagnostic purposes, radionuclide applications can be expanded using alpha- or beta-emitting isotopes that have therapeutic effects in the (surroundings of) cells expressing the target of the antibody used.

The term ‘theranostics’ is a combination of therapy and diagnostics. It relates the combined use of diagnostic (such as gallium-68, zirconium-89, fluorine-18) or therapeutic (such as actinium-225, lutetium-177, rhenium-186/188) radioisotopes coupled to a cancer-specific targeting vector. Exciting developments in the field of theranostics include ^177^[Lu]Lu-prostate specific membrane antigen (PSMA) [11, 12] and the increasing use of ^177^[Lu]Lu-DOTATATE [13]. Both drugs have resulted in prolonged overall survival and an added clinical benefit for patients, including an acceptable toxicity profile compared to the comparator treatment.

This narrative review discusses the possibilities of theranostics currently available and under development in breast cancer. We provide an overview of diagnostic radiotracers that represent targets of breast cancer therapies, such as HER2, PD-L1, ER and PARP. In addition, we will discuss developments of radiopharmaceutical therapy for breast cancer management.

## Main text

**Fig. 2:**
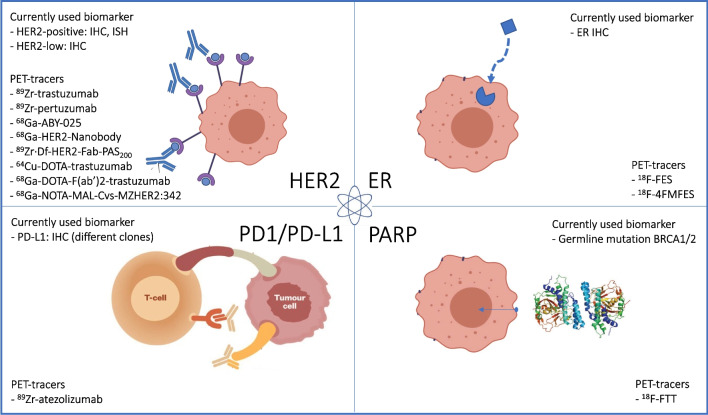
Overview of treatment targets in breast cancer used in current clinical practice and available diagnostic radiotracers, where imaging studies in humans with breast cancer have been performed

In this review, we will cover the following treatment targets: HER2, to select for HER2-targeted agents; PD-L1, used to select for checkpoint inhibitors; and ER, for antihormonal drugs. In addition, we included PARP, where patients are selected for treatment with PARP inhibitors (PARPis) based on the assessment of mutation in the breast cancer genes 1 and 2 (BRCA1 and BRCA2) (Fig. 2).

Other targeted therapies currently used in breast cancer management include the antibody–drug conjugate sacituzumab govitecan in metastatic triple-negative breast cancer (mTNBC [14]) targeting trophoblast cell surface antigen 2 (Trop2) and the phosphoinositide 3-kinases (PI3Ks) inhibitors for patients with ER + tumors with a somatic mutation in PIK3CA [15]. The latter two will not be discussed in this review; for sacituzumab govitecan, no approved and/or clinically used predictive biomarker has been identified [13, 16]. For the PI3KC inhibitors, up to the best of our knowledge, only one preclinical study with the radiotracer [^11^C]C-pictilisib that was investigated in tumor-bearing mice has been published so far [17].

### Diagnostic tracers for human epidermal growth factor receptor 2 (HER2) expression

#### Relevance

The clinical relevance and solid prognostic value of overexpression of the HER2 receptor were discovered almost four decades ago [18]. Pivotal adjuvant trials such as HERA and BCIRG-006 in patients with HER2-overexpressing breast cancer paved the way for numerous studies with HER2-targeted drugs in different cancer patient populations [19]. Currently, anti-HER2 therapies are approved using various types of drugs (monoclonal antibodies, tyrosine kinase inhibitors and antibody drug conjugates [ADC]) in various tumor types with overexpression, gene amplifications and mutations of the HER2 receptor.

For breast cancer, HER2-targeted drugs are indicated for treating HER2-positive breast cancer, i.e., cancer with a high degree of HER2 expression. Moreover, the HER2-targeted ADC trastuzumab deruxtecan (TDXd) has recently been approved for breast cancer patients with tumors that are formally not HER2-positive but so-called ‘HER2-low’ [20]. In the DESTINY-Breast04 study, an improvement in overall survival by TDXd versus physicians’ choice of chemotherapy was found in patients with HER2-low metastatic breast cancer [21–23].

Heterogeneity in HER2 status between and within metastases and receptor conversion over time has been reported in several breast cancer patient cohorts [9]. Reliable assessment of HER2 status and variations in formal HER2-positive lesions have become even more relevant after the identification of the clinical relevance of the HER2-low subgroup, which is estimated to constitute ~ 50% of all breast cancer patients [20].

#### Evidence from clinical studies

Several radiopharmaceuticals targeting HER2 have been developed and tested in breast cancer patients, both tracers for imaging with PET and with single-photon emission tomography (SPECT) [24]. Due to the lower image quality and lower resolution, SPECT scans are nowadays sparsely used. PET imaging tracers used to identify HER2-positive lesions include radiolabeled monoclonal antibodies ([^89^Zr]Zr-trastuzumab, [^89^Zr]Zr-pertuzumab [25, 26]) and smaller HER2-specific affinity proteins ([^68^Ga]Ga-ABY-025, [^68^Ga]Ga-HER2-nanobody and [^68^Ga]Ga-DOTA-F(ab’)2-trastuzumab) [27, 28].

Table 1 summarizes the different tracers that have been studied up to now, ^89^Zr-trastuzumab being the one that has been evaluated most and with the most important results to date.

**Table 1:** Overview of clinical trials in breast cancer patients aimed at molecular imaging of targets of treatment: HER2, ER, PD-L1 and PARP

Target	Traces	Population	Primary aim	Results
**HER2**	^89^Zr-trastuzumab	14 pts, HER2 + mBC [25]	Dosing of tracer and timing of image acquisition	Relative uptake values in different organs, in tumor lesions and healthy tissue
		56 pts HER2 + , mBC [29]	Investigate heterogeneity in HER2 status and evaluate therapy-predictive role for T-DM1	Heterogeneity: 29% had negative PET, 46% heterogeneous uptake Predictive role: compared to RECIST 1.1, NPV and PPV for HER2-PET were 88% and 72% Combining HER2- and FDG-PET strongest NPV and PPV (both 100%)
		11 pts, HER2- eBC [30]	Investigate whether HER2-PET can identify HER2-positive lesions in previously known HER2-negative tumors	Four patients with uptake on HER2-PET, of which one was confirmed as HER2 + on biopsy. The other 3 were considered as having false-positive HER2-PET results
	^64^Cu-DOTA-trastuzumab	8 pts, HER2 + mBC [31]	To detect and measure tumor uptake of trastuzumab	A cold dose of trastuzumab improves image quality High sensitivity of visualizing HER2 + lesions
		18 pts mBC, 2 HER2-PET scans [32]	Compare HER2 status on PET (SUV) and biopsy (ISH)	PET uptake strongly related to pathological HER2 status, but with large interpatient variability
		6 pts, HER2 + eBC or mBC [33]	Safety, distribution, internal dosimetry	Feasible for the identification of HER2-positive lesions. Acceptable dosimetry and pharmacologic safety
	^89^Zr-pertuzumab	6 pts, HER2 + mBC [26]	First in human	Safety, dosimetry, biodistribution and successful HER2-targeted imaging
		24 pts mBC, HER2-neg primary tumor [34]	Evaluate HER2-positive lesions in pts with HER2-neg tumors	6/24 pts with 89Zr-pert avid lesions, of which 3 have biopsy-confirmed HER2 positivity
		9 pts, HER2- eBC [26]	Study heterogeneity in HER2 status in patients with HER2-negative primary tumor	5 patients with HER2-uptake on PET; of these, two had biopsy-proven HER2-positive metastases. In the other three, tumor biopsy revealed HER2-negative status, and PET findings were considered false positive
	^68^Ga-ABY-025	16 pts, HER2 + mBC [27]	Phase I/II aimed to study the effect of tracer peptide mass, test–retest variability and correlation of quantified uptake with histopathology	Correlation PET-SUV with IHC 0.91, no false-positive tracer uptake in HER2- lesions, SUV 5 times higher in HER2 + vs HER lesions, test–retest intra-class correlation r = 0.996
	^68^Ga-DOTA-F(ab’)2-trastuzumab	16 pts mBC, HER2 + and HER2- [35]	First clinical evaluation to study safety, pharmacokinetics, biodistribution and dosimetry profile	Safety was confirmed, no unexpected findings related to dosimetry and distribution. Tumor uptake was seen in 4 out of 8 pts with HER2 + mBC
	^89^Zr∙Df-HER2-Fab-PAS200	1 pt, HER2 + mBC [36]	First clinical study	Image acquisition after single dose, lesions could be detected 24 h after injection after appropriate blood clearance
	^68^Ga-HER2-Nanobody	20 pts with HER2 + eBC or mBC [37]	Phase 1	Safe. Clear uptake in metastatic lesions, variable uptake in primary breast tumors
	^68^Ga-NOTA-MAL-Cvs- ZHER2:342	2 pts mBC, one HER2 + and one HER2- [38]	Phase 1	SUVmax HER2 + tumors 2.16 ± 0.27), SUVmax HER2- tumors 0.32 ± 0.05
**PD-L1**	^89^Zr-atezolizumab	4 pts with mTNBC [39]	First In Human study	Tracer uptake in metastatic lesions in all mTNBC patients, no unexpected safety signals
**ER**	^18^F-FES	90 pts with relapsed or de novo metastatic BC [40]	Assess the diagnostic accuracy and safety of 18F-FES-PET/CT to assess ER status	FES-PET positive: 36/36 biopsied with ER + tumor FES-PET negative: 11/49 biopsied with ER + tumor SUV_max_^18^F-FES vs Allred score r = 0.83, P < 0.0001
		19 pts with relapsed BC [41]	Investigate the utility of FES-PET to predict overall response to first-line endocrine therapy and validate previously defined cut-off SUVmean > 1.5	FES SUVmean dynamic vs response to therapy after 6 months: partial response mean SUVmean 2.2 (1.8–3.0); stable disease SUVmean 3.6 (1.9–5.7); progressive disease SUVmean 1.9 (0.2–3.1)
		Meta-analysis of 12 trials [42]	Assess lesion-level agreement between ER IHC assays and qualitative assessment by 18F-FES-PET, primary analysis focused on metastatic lesions	Sensitivity metastatic lesions 0.78 (95% CI 0.65–0.88), specificity 0.98 (95% CI 0.65–1)
		Prospective trial 200 pts mBC [43]	ER expression in the biopsied metastasis was related to qualitative whole-body ^18^F-FES-PET evaluation and quantitative ^18^F-FES uptake in the corresponding metastasis	In 200 pts: sensitivity 95% (95% CI 89–97%), specificity 80% (95% CI 66–89%)
	^18^F-4FMFES	Prospective trial 31 BC pts ER + tumors [44]	Compare diagnostic potential of 18F-4FMFES vs 18F-FES	2.5-fold increase in metabolic stability of ^18^F-4FMFES over ^18^F-FES. SUV_max_ similar for both tracers, but improved diagnostic performance related to lower uptake in non-specific tissues for ^18^F-4FMFES
**PARP**	^18^F-FTT	Prospective trial in 13 BC pts with PET before and after PARPi treatment [45]	Evaluate in vivo visualization of PARP inhibitor pharmacodynamics, relate blockade of PARP1 expression in vitro or with repeated in vivo imaging	Nine of 13 pts underwent tumor resection and in vitro evaluation of ^18^F-FTT uptake with ^125^I-KX1 (an analog of ^18^F-FTT and uptake was blocked with PARPi Of the other 4 patients, 3 had ^18^F-FTT PET uptake pretreatment, and all had uptake blocked with treatment with a therapeutic PARPi as seen on a second ^18^F-FTT PET
		30 pts with untreated stage I-IV BC [46]	Evaluate PARP1 expression in relation to tumor subtype and BRCA mutation status	Overlapping ranges of SUV_max_ between different tumor subtypes and between patients with and without germline BRCA mutations

#### Current applicability

Up to now, various HER2-targeting tracers have been tested in clinical trials in breast cancer patients. All of those are small to moderately sized patient populations, and the prime focus of these studies has been to investigate safety, feasibility and correlation with HER2 status on a tumor biopsy. The ZEPHIR trial is the only one that has investigated the therapy-predictive role of [^89^Zr]Zr-trastuzumab for treatment with the ADC trastuzumab emtansine [29]. In this prospective multicenter trial of 56 patients with HER2-positive mBC, a combination of pretreatment HER2 imaging and early FDG-PET/CT was found to accurately predict morphological treatment response, leading to the conclusion that targeted HER2 imaging could be of great value both for the understanding of tumor heterogeneity and function as an aid in the selection of patients that would benefit from targeted treatment. While this may certainly be the case for trastuzumab emtansine, the much stronger bystander effect of recent ADCs such as TDXd may cause difficulties in using HER2-targeted imaging for the prediction of treatment response, a potential issue which should be evaluated further in future trials.

Of note, one study with [^89^Zr]Zr-trastuzumab [47] and one with [^89^Zr]Zr-pertuzumab [26] showed a number of false-positive PET results for HER2 status. It is with the current knowledge of HER2-low tumors unknown whether these metastases nowadays might have classified as HER2-low tumors.

#### Ongoing studies with diagnostic HER2-tracers

Several clinical trials with HER2-PET imaging are ongoing, aimed at establishing the role of HER2-PET imaging as a biomarker for treatment selection. In the Affibody-3 trial (NCT03655353), the Affibody molecule based on [^68^Ga]Ga-ABY-025 is used for non-invasive quantification of HER2 expression in patients with primary and metastatic breast cancer, with the primary aim to study the correlation between the HER2 expression measured by [^68^Ga]Ga-ABY-025 PET and standard histopathology. A metabolic response (based on findings from [^18^F]F-fluordesoxyglucose [FDG]-PET) activity after anti-HER2 treatment is included, and in an interim analysis of 40 patients presented at the San Antonio Breast Cancer Conference 2022, it was found that tracer uptake could predict metabolic response to treatment better than conventional IHC and that the tracer may be useful as an adjunct diagnostic tool. [^68^Ga]Ga-ABY-025 is now being investigated for its ability to detect HER2-low metastatic breast cancer at our institution, where patients with HER2-low metastatic breast cancer will undergo one HER2-PET followed by tumor biopsies (NCT05619016). Furthermore, the closely related HER2-specific tracer [^18^F]F-GE-226 is being investigated in the HERPET study (NCT03827317). The trial determines the uptake in tumors and healthy tissues of [^18^F]F-GE-226 to compare the difference between patients with HER2-positive and HER2-negative lesions. An extended trial has been initiated that will further investigate basic properties of the tracer, here called [^18^F]F-GEH121224, to guide further clinical development.

Another type of tracer molecule is the single variable domain of a heavy-chain (VHH) antibody specific for HER2. After a successful initial study, two continued studies have now been initiated with [^68^Ga]Ga-NOTA-Anti-HER2 VHH1. In the VUBAR study (NCT03924466), image-based HER2 quantification repeatability will be investigated, and one cohort will undergo [^68^Ga]Ga-NOTA-Anti-HER2 VHH1 PET/CT before and after start of neoadjuvant treatment to study potential for added value of HER2 imaging in the neoadjuvant setting. The second study is an evaluation of uptake of the tracer in brain metastases in breast cancer patients (NCT03331601), where a change in uptake in brain lesions in response to treatment will be assessed.

Imaging of HER2 was pioneered using the HER2-specific antibody trastuzumab, and trials are still investigating scientific questions using this tracer, e.g., to define which patients are likely to respond to targeted HER2 agents using [^89^Zr]Zr-trastuzumab (NCT03321045) or exploring a different PET radionuclide [^64^Cu]Cu-DOTA-trastuzumab (NCT05376878). Also, other antibodies are investigated, such as the site-specifically labeled [^89^Zr]Zr-ss-pertuzumab (NCT04692831).

### Diagnostic tracers for programmed death ligand 1 (PD-L1) expression

#### Relevance

Currently, two checkpoint inhibitors are approved for breast cancer patients and used in clinical practice. The European Medicines Agency (EMA) and Federal Drug Administration (FDA) approved the programmed death receptor 1 (PD-1) antibody pembrolizumab for the treatment of patients with early triple-negative breast cancer (TNBC) who are receiving neoadjuvant systemic therapy and for patients with advanced TNBC whose tumors express PD-L1 (graded as combined positive score, CPS ≥ 10) by means of the PD-L1 IHC 22C3 pharmDx test. Atezolizumab, a PD-L1 antibody, is approved by the EMA for treating patients with advanced or metastatic TNBC with PD-L1 expression in at least 1% of immune cells with the SP142 Ventana antibody.

Extensive research efforts have searched for the optimal therapy-predictive biomarker for checkpoint inhibitors in solid tumors. Up to now, the only approved biomarker is PD-L1 expression on a tumor biopsy according to pre-defined criteria mentioned above [48]. However, this method also has a suboptimal performance, as concluded in a systematic review and meta-analysis across all solid tumors [49]. For TNBC, several alternative candidate biomarkers have been proposed but have yet to be validated thoroughly enough to be implemented in clinical practice and replace PD-L1 IHC on a tumor biopsy [50]. It has been unequivocally described that PD-L1 expression can change over time from primary to metastatic breast cancer and have varying levels of expression in metastases in different organs [8]. This further limits the therapy-predictive role of PD-L1 IHC for the benefit of checkpoint inhibitors for patients with TNBC. Interestingly, PD-L1 status in the primary tumor has no therapy-predictive role for adding pembrolizumab to a chemotherapy backbone in the neoadjuvant treatment setting, as was observed in the Keynote-522 trial [4].

#### Evidence from clinical studies

A few radiotracers representing targets of checkpoint inhibitors, or their downstream intracellular effects, have been studied [51] in the first-in-human (FIH) study with [^89^Zr]Zr-atezolizumab in 25 patients (NCT02453984), including four patients with mTNBC [39]. This study showed that the tracer was feasible and safe. The results indicated clear expression heterogeneity within and between lesions and a promising role as a predictive marker for response to treatment with PD-L1 antibodies. For the patients with mTNBC in this study, the maximum standardized uptake value (SUV_max_) was clearly over the SUV_mean_ in the blood pool measured over the aorta, with varying levels of SUV_max_ in different organs. Higher SUV_max_ was related to a better antitumor response, according to RECIST. To the best of our knowledge, this is the only published study on non-invasive imaging with a PD-L1 tracer in patients with breast cancer.

Several preclinical studies have reported warranting results, e.g., with the anti-PD-L1-B11 clone antibody coupled to zirconium-89 [52], [^89^Zr]Zr-Df-bintrafusp-alfa and [^89^Zr]Zr-avelumab [53–55].

#### Current applicability

PD-L1 IHC on a tumor biopsy (either from the primary tumor or a metastatic lesion) is the only approved biomarker for adding checkpoint inhibitors in mTNBC. There is, up to now, minimal evidence from clinical trials in patients that reveal the therapy-predictive and tumor biological value of molecular imaging with PD-L1 targeted tracers in patients.

#### Ongoing studies with diagnostic PD-L1 tracers

Several other radiolabeled PD-L1 and PD-1 antibodies are currently tested in clinical trials, for example, ^89^Zr-pembrolizumab (active trials: NCT02760225, NCT03065764), [^89^Zr]Zr-durvalumab (NCT03610061, NCT03829007) and [^89^Zr]Zr-avelumab (NCT03514719). We have not identified other clinical trials in patients with mTNBC or other biological subtypes of breast cancer where the role of non-invasive PD-L1 PET imaging is investigated.

Currently, only one trial in breast cancer patients with this radiotracer has been registered at clinicaltrials.gov, one in patients with metastatic lobular breast cancer treated with carboplatin and atezolizumab (NCT04222426). This study was recently terminated after including one patient in the main trial, and a parallel biomarker imaging substudy was closed.

At our institution, we are initiating a clinical trial for patients with metastatic or irresectable TNBC who undergo a baseline [^89^Zr]Zr-atezolizumab PET/CT and a tumor biopsy for PD-L1 IHC before starting with first-line systemic therapy. In this trial (NCT05742269), patients with PD-L1-positive tumors according to PD-L1 PET and/or IHC will receive atezolizumab with a chemotherapy backbone (carboplatin and nab-paclitaxel). The primary endpoint of this PD-L1 PET trial is the statistical agreement between PD-L1 assessed by IHC and PET by estimating a kappa coefficient.

### Diagnostic tracers for estrogen receptor (ER) expression

#### Relevance

Anti-hormonal drugs have been a cornerstone of systemic breast cancer therapies for decades. Around 70% of breast cancers express ER. ER expression is a highly reliable and systematically used predictive biomarker for treatment with antihormonal therapy [10]. Different antihormonal drugs are available, the largest groups being selective estrogen receptor modulators (SERM) and steroidal/non-steroidal aromatase inhibitors. Changes in ER expression over time, heterogeneity within and between tumors and ER mutations are known complicating factors [9, 56], underscoring the vital need to obtain representative and actual proof of ER status before antihormonal therapies are initiated.

#### Evidence from clinical studies

A few ER-targeted imaging tracers have been studied, most of them with the FDA-approved 16α-[^18^F]F-fluoro-17β-estradiol ([^18^F]F-FES) tracer [57]. Other ER-targeting tracer studies include 4-fluoro-11β-methoxy-16α-[^18^F]F-fluoroestradiol ([^18^F]F-4FMFES) [44] exhibiting a sensitivity of 95% (89 to 97), a specificity of 80% (66 to 89), a positive predictive value of 93% (87 to 96) and a negative predictive value of 85% (72 to 92) in predicting ER IHC.

#### Current applicability

The use of [^18^F]F-FES-PET is supported by the strongest evidence in targeted nuclear tracers so far, with a meta-analysis supporting the reliability of tracer uptake to predict ER status using IHC on a tumor biopsy. Several limitations have so far impeded a more general use of FES-PET to assess ER status in a patient with metastatic breast cancer. The most apparent reason is the need for increased availability of the tracer and local image acquisition experience and protocols, even when PET camera facilities are present. The washout of prior ER antagonists is a special consideration for [^18^F]F-FES-PET imaging. Because [^18^F]F-FES-PET measures the regional binding of estrogens to the estrogen receptor, exposure to SERDs will block ER and thereby prevent tracer accumulation leading to a [^18^F]F-FES-negative lesion. For this reason, a 6-week washout is indicated for tamoxifen and fulvestrant. This phenomenon is not present in prior treatment with aromatase inhibitors.

In the recently published meta-analysis presenting the results of the IMPACT-trial, the authors present a flowchart for the work-up of patients with metastatic breast cancer, including the role of [^18^F]F-FES-PET in guiding treatment decisions [43].

An unresolved challenge in establishing the role of [^18^F]F-FES-PET in clinical practice is the need for more evidence regarding the therapy-predictive role of ER status and how the imaging results can guide systemic therapies. Due to disease heterogeneity, it seems rational to integrate a quantitative component in reviewing an [^18^F]F-FES-PET scan to describe the overall ER status of the metastases found on CT.

Based on the current evidence, FES-PET can be preferentially used in clinical practice when a biopsy is not feasible or not wanted, for instance, at a later disease stage. Nevertheless, a tumor biopsy will remain the cornerstone of the work-up in metastatic breast cancer since [^18^F]F-FES-PET alone will not be able to inform the clinician about additional tumor biological factors such as histology and/or HER2 status.

#### Ongoing studies with diagnostic ER-tracers

Currently, around 12 trials incorporating imaging with [^18^F]F-FES are ongoing (search in Clinicaltrials.gov, accessed November 21, 2022). Several interesting research questions that are being evaluated in these trials are, among others, the establishment of a pharmacokinetic model and validation of quantitative parameters for clinical practice (NCT05088785); the therapy-predictive value of FES-PET for the efficacy of first-line antihormonal treatment for ER + metastatic breast cancer (NCT02398773); and the combined assessment of FDG-PET and FES-PET in the management of both early and metastatic breast cancer (NCT04692103). In the SONIA trial, which investigates the optimal timing of adding CDK4/6 inhibitors to antihormonal drugs, an imaging substudy SONImage is performed where FES-PET is done at baseline prior to start of systemic treatment (NCT04125277).

### Diagnostic tracers for polyadenosine diphosphate-ribose polymerase (PARP) expression

#### Relevance

The nuclear enzyme PARP1 is central in sensing DNA damage and facilitating repair. Tumors with BRCA1/2 mutations are highly dependent on PARP1 as an alternative DNA repair mechanism. PARPis generate synthetic lethality in tumors with BRCA mutations, resulting in cell cycle arrest and apoptosis [58]. PARPis have proven clinical efficacy for breast cancer management and are approved for use in early and metastatic disease settings for patients with germline mutations in the BRCA1/2 genes. In the OlympiA trial, the efficacy of 1-year treatment of olaparib versus placebo in the adjuvant setting was investigated in patients with early HER2-negative breast cancer who had received (neo)adjuvant chemotherapy, surgery, antihormonal and radiation therapy if indicated. In the olaparib group, overall survival was significantly improved compared to the placebo, with a four-year overall survival of 89.8% in the intervention group versus 86.4% in the placebo group (Δ 3.4%, 95% CI − 0.1% to 6.8%) [5, 59]. For patients with germline BRCA1/2 mutations with metastatic HER2-negative breast cancer, treatment with the PARPis olaparib or talazoparib is associated with more prolonged progression-free survival compared to regular chemotherapeutic treatments [60, 61].

There is a strong biological rationale to assume that the benefit from PARPis is likely not to be confined to patients with germline BRCA1/2 mutations but that this may exist even in those with other defects of homologous recombination (HRD) [62].

Theoretically, PARP1 overexpression should be the best and direct predictive biomarker for PARP1 inhibitors. To date, PARP expression and BRCA status in tumors can be assessed by IHC or genetic sequencing on biopsy samples. However, the results of ICH of PARP1 expression have demonstrated mixed results, suggesting inconsistency of staining procedures [63] with lack of a validated staining protocol that could be applied in clinics. In addition to the time-consuming pathological procedures, such analyses' results depend on the representativity and quality of tumor biopsy samples. Therefore, PET imaging of PARP expression is a candidate predictive biomarker to select patients that could benefit from PARPis. In addition, radiolabeled PARPis could also be used to characterize dynamic changes in tumoral PARP expression during treatment with PARPis or DNA-damaging agents.

#### Evidence from clinical studies

Several radiopharmaceuticals targeting PARP have been developed and tested in phase 1 studies, assessed with either intra-operative optical imaging or PET imaging [64]. The first PARP-tracer studied is [^18^F]F-fluorthanatrace (FTT), which was evaluated in a FIH study in eight patients with various solid malignancies [65].

A prospective non-randomized clinical trial of 30 participants with early and locally advanced breast cancer studied the correlation of [^18^F]F-FTT uptake in different breast cancer subtypes [66]. The SUV from these patients ranged from 2.6 to 11.3 g/mL, independent of cancer subtypes and germline BRCA 1/2 mutation status. The SUV varied greatly in patients with BRCA1/2 pathogenic variants, and the range overlapped values from patients without BRCA 1/2. In this preliminary study, patients with mutations in BRCA1/2 genes showed lower levels of tracer uptake than patients who retained loss of heterozygosity. These results showed that the level of radioligand binding varied considerably across and within investigated breast cancer subtypes, including germline or tumor mutations in BRCA-related genes.

In a follow-up study in patients with mBC receiving PARPi therapy, [^18^F]F-FTT uptake in known sites of disease was blocked by PARPi treatment [45]. Drug PARP1 occupancy was also measured by autoradiography radioligand-binding studies of cancer tissue samples using [^125^I]I-KX1 with and without pharmacologic levels of olaparib. [^125^I]I-KX1 binding was suppressed by greater than 80% by olaparib and matched PARPi blockade measured at pre- and post-PARPi PET. Despite the small sample size (four patients; age range, 41–71 years; median age, 52 years; all women; stage III or IV breast cancer), this study demonstrates the potential of [^18^F]F-FTT PET to non-invasively quantify PARP1 expression and provides early evidence of using this modality to assess PARPi drug-target engagement, indicating its potential as a biomarker for treatment with PARPis.

Other phase I trials have indicated the safety and feasibility of visualizing tumors/quantifying PARP expression by means of [^18^F]F-PARPi in a range of tumor types (excluding breast cancer), for example, in patients with ovarian cancer [67] and head-and-neck cancer [68]. In a preclinical model, the tracer [^18^F]F-olaparib showed successful uptake on PET imaging in mice with xenografts overexpressing PARP1 [69].

#### Applicability

The results of such studies could be used to identify rational therapy selection with PARPis to maximize the therapeutic benefits while minimizing exposure to toxicities in patients who would not respond, apart from the currently used selection based on germline mutations in BRCA 1/2. Radiolabeled PARPis could also be used to characterize dynamic changes in tumoral PARP expression during treatment with PARPis or DNA-damaging agents, thereby enhancing tumor biological understanding and providing a rationale for the combination of PARPis with other drugs.

### Therapeutic radionuclides for breast cancer management

#### Current status

For several decades, palliative treatment with bone-seeking isotopes such as radium-223, strontium-90 and samarium-153 ethylenediamine tetra-methylene phosphonic acid (EDTMP) has been used to alleviate symptoms from painful bone metastases [70]. Due to potent analgesics and other palliative treatment options, such as external beam radiotherapy, these are sparsely used nowadays and no longer routinely incorporated into clinical practice guidelines [10, 46].

#### Clinical studies with radionuclide treatment for breast cancer: a theranostic approach

The concept of targeted delivery of radioisotopes coupled with tumor-specific antibodies is now increasingly being explored in breast cancer management. This approach is in its very beginning, and very few studies in humans are published. Targets of interest include HER2 and new tumor-specific targets, as discussed below.

Up to now, one phase I study in six healthy volunteers and three patients with HER2-positive metastatic breast cancer has been reported with Iodine131 coupled to a HER2 antibody fragment: [^131^I]I -GMIB-anti-HER2-VHH1 [71]. No drug-related adverse events were observed in any of the nine subjects, and tracer uptake was noted in metastatic lesions in the breast cancer cohort. An expanded cohort with a phase I/II dose escalation study with the same product is ongoing in patients with metastatic HER2-expressing breast, gastric and gastroesophageal cancer (NCT04467515); the primary outcome is the therapeutic efficacy of the experimental drug. Other HER2-targeted therapeutic isotopes that are currently trialed in early phase clinical trials include a thorium-227-coupled HER2 antibody BAY2701439 for patients with HER2-expressing breast and gastroesophageal cancers that have progressed on earlier HER2-targeted treatment lines (NCT04147819).

Another alpha-therapy antibody is in a phase 1/2 study of [^225^Ac]Ac-FPI-1434. The antibody targets insulin growth factor 1 receptor (IGF1R) and is currently being tested in patients with tumors that express IGR1R, including breast cancer (NCT03746431).

### New targets of interest for breast cancer theranostics

Radiotracers representing other targets of potential interest in breast cancer management include [^68^Ga]Ga-DOTATATE and [^68^Ga]Ga-PSMA; for both targets, preliminary work confirmed tracer uptake in metastatic lesions in patients with metastatic breast cancer [72, 73]. This points to the potential role of therapeutic application of somatostatin receptor 2 (SSTR2)- or PSMA-labeled radiopharmaceuticals and provides a rationale for a theranostics approach where diagnostic imaging is performed and, when the target of treatment is present, a therapeutic isotope is considered as a next systemic treatment option.

Additional cancer-specific targets are explored for imaging, such as gastrin-releasing peptide receptor (GRPR) imaging in breast cancer patients with ER-positive tumors using the receptor antagonist [^68^Ga]Ga-RM26 [74]. Immune activation and T-cell recruitment induced by immune checkpoint inhibitors can be visualized by several other PET tracers [51], a process which was recently studied in detail using the [^89^Zr]Zr-ED88082A tracer in patients with different metastatic solid malignancies, including breast cancer [75].

An interesting concept that warrants further exploration is dual-imaging tracers, which will enable a more refined disease characterization, for example, through combined assessment of ER and HER2. Such tracers have, up to now, only been evaluated in preclinical models [76].

## Discussion

The concept of an individualized treatment through theranostic tools might be an old one [77], but the continuous advances in molecular imaging create new possibilities and open new research fields examining the clinical use of theranostics. The main application of theranostics in breast cancer today is focused on improving patient selection for targeted therapies on the basis of specific molecular features of disease, but also to objectively monitor response of such therapies. Future developments are anticipated, especially in the development of therapeutic radiopharmaceuticals.

Despite the progress, several caveats should be taken into account in interpreting the data discussed in this review. Firstly, the fact that a tumor accumulating a radiotracer does not automatically ensure the therapeutic functionality of the radiolabeled drug. The present review is focused on radiolabeled *targeted* drugs. PET imaging with such tracers visualizes the delivery of the drug to the target. However, how functional this drug will be depends on several factors including the drug’s mechanism of action.

Secondly, tumor heterogeneity is a very important issue to address. It is expected that molecular imaging with targeted drugs can show heterogeneity in tracer uptake between primary tumor and its metastases, or between different metastases in the same patient—a phenomenon that is repeatedly reported for pathological analyses on a tumor biopsy [8, 9]. At the same time, due to the intrinsic resolution limitations of molecular imaging it is not yet known whether it is possible to assess biological heterogeneity in the level of cell and even tissue.

Thirdly, very few studies have focused on the clinical utility and validity of different radiotracers. Several tracers have until now been tested for the safety, feasibility to detect the target and their accuracy in visualizing the target, e.g., very early phase of clinical trials, often with a small number of included patients. This question of the clinical utility of baseline HER2-PET was prospectively evaluated in the ZEPHIR study described above, with an attempt to identify patients most likely to benefit from T-DM1 [29].

Another item to consider when interpreting the findings from studies included in this review is that these have almost exclusively been performed as single-center studies, which raises questions about the reproducibility of these imaging tests when performed at other sites. Likewise, definitions on a ‘positive’ and a ‘negative’ test differ significantly between the studies, and for future application, in clinics robust tools based on combined data from imaging warehouses will be desirable [47].

Last, studies examining the correlation of tracer uptake with biological characterization of the tumors are very limited and usually based on quantification by SUV, which is presumably not scientifically correct when antibodies are studied. New approaches including parametric imaging and AI could be a step in right direction.

### Future directions

Technical developments are expected to enhance the clinical validity of and access to radiotracers that are feasible for use in daily practice. There is a broad spectrum of targeting vectors that are used for imaging, spanning from full-length monoclonal antibodies, diabodies, minibodies, antibody fragments and peptides to small molecules [78]. While antibodies are well suited for therapeutic applications, small-sized fragments or molecules are better suited for imaging purposes. A major advantage of the small-sized molecules coupled to isotopes with a relatively short half-life is that high-contrast images can be acquired shortly after administering such tracers to the patients, due to the fast tumor penetration and rapid excretion. In contrast, for large antibodies that, because of their slow accumulation in the tumors, must be labeled with long-lived isotopes (such as zirconium-89), useful image acquisition cannot be done earlier than 5–7 days after tracer injection [79]. Hence, there are substantial research efforts in the development of small-sized tracers for molecular imaging including their role for therapy prediction of monoclonal antibodies [80]. It is, however, for practical and logistical reasons, not mentioning the economical aspects, possible to directly radiolabel ADCs for imaging with the purpose of possibly predicting ADC’s therapeutics effects.

Routine testing for therapy-predictive biomarkers usually includes different targets, e.g., HER2, ER and PD-L1, in the case of breast cancer treatment selection. The development of radiotracers that represent several characteristics of tumors through bispecific tracers might be an elegant solution, where such tracers have been tested in animal models and may reach human studies within the nearest decade [53, 81].

## Conclusions

Molecular imaging with radiotracers that represent targets of (breast) cancer treatments holds promise as a precision medicine tool that enables a minimally invasive, real-time, whole-body assessment of the presence of the target of treatment. New tracers enable same-day imaging with few manageable side effects. A primary research focus entails establishing the therapy-predictive value of tracer uptake for drugs targeting the tumoral feature that is visualized. Ongoing clinical trials will establish the role of therapeutic radionuclides for breast cancer management, with radiopharmaceuticals targeting established targets or treatment as well as expanded disease features. These efforts will pave the way for implementing theranostics in breast cancer management.

## Data Availability

No data were generated during this work.

## Abbreviations

ADC: Antibody drug conjugate
BRCA1, BRCA2: Breast cancer genes 1 and 2
EMA: European Medicines Agency
ER: Estrogen receptor
EDTMP: Ethylenediamine tetra-methylene phosphonic acid
FIH: First in human
FDG: Fluordesoxyglucose
FTT: Fluorthanatrace
HER2: Human epidermal growth factor receptor 2
IHC: Immunohistochemistry
ISH: In situ hybridization
IGF1R: Insulin growth factor 1 receptor
mTNBC: Metastatic triple-negative breast cancer
PI3Ks: Phosphoinositide 3-kinases
PARP: Polyadenosine diphosphate-ribose polymerase
PET: Positron emission tomography
PD-1: Programmed death receptor 1
PSMA: Prostate specific membrane antigen
PD-L1: Programmed death ligand 1
SERM: Selective estrogen receptor modulators
SPECT: Single-photon emission tomography
SSTR2: Somatostatin receptor 2
SUV: Standardized uptake value
TDXd: Trastuzumab deruxtecan
Trop2: Trophoblast cell surface antigen 2
